# Beef Quality Classification with Reduced E-Nose Data Features According to Beef Cut Types

**DOI:** 10.3390/s23042222

**Published:** 2023-02-16

**Authors:** Ahmet Feyzioglu, Yavuz Selim Taspinar

**Affiliations:** 1 Department of Mechanical Engineering, Marmara University, Istanbul 34722, Turkey; 2 Doganhisar Vocational School, Selcuk University, Konya 42930, Turkey

**Keywords:** decision support system, e-nose, data fusion, control, beef quality

## Abstract

Ensuring safe food supplies has recently become a serious problem all over the world. Controlling the quality, spoilage, and standing time for products with a short shelf life is a quite difficult problem. However, electronic noses can make all these controls possible. In this study, which aims to develop a different approach to the solution of this problem, electronic nose data obtained from 12 different beef cuts were classified. In the dataset, there are four classes (1: excellent, 2: good, 3: acceptable, and 4: spoiled) indicating beef quality. The classifications were performed separately for each cut and all cut shapes. The ANOVA method was used to determine the active features in the dataset with data for 12 features. The same classification processes were carried out by using the three active features selected by the ANOVA method. Three different machine learning methods, Artificial Neural Network, K Nearest Neighbor, and Logistic Regression, which are frequently used in the literature, were used in classifications. In the experimental studies, a classification accuracy of 100% was obtained as a result of the classification performed with ANN using the data obtained by combining all the tables in the dataset.

## 1. Introduction

Meat, which is one of the richest food sources in terms of protein and fat and is frequently consumed by living things in nature, is a product that should be consumed in a short time. It should be stored in cold and sterile environments so that its quality does not deteriorate in the process from production to consumption [1]. The spoiling time of the meat may differ depending on the creature from which it is obtained. The quality of meat is a factor that is directly related to its nutritional value and economic value. Therefore, quality controls of meat must be carried out carefully in all processes from production to consumption. Quality features include the physical and chemical features of meat [2]. Since meat is an attractive food source for microorganisms, microbial growth also occurs rapidly in meat, which is a factor impacting its flavor, smell, and nutritional value [3]. Meet quality controls are carried out by experts; however, problems can arise since it is not always possible to find such specialists.

Automatic detection systems continue to be developed in order to maintain the necessary controls and evaluations for when experts are unavailable [4]. As food spoils, odor and gas are emitted due to microbial activity [5]. Here, the odor is caused by the different gases that spread, and it is possible to obtain information about the quality of food by detecting these emitted gases through sensors [6,7]. These systems, which contain a sensor array and imitate the human nose, are called electronic noses (e-noses). Electronic noses are used in detecting the freshness of meat [8], drug detection [9], disease diagnosis [10], and the diagnosis of the COVID-19 infection, which has turned into a pandemic in recent years [11].

There are many studies in the literature that use data from e-noses to automatically determine the quality of different foods [7,12,13,14]. E-nose studies tracing perishable foods are more common [15,16]. In order to clarify the aim of this study, the studies conducted with e-nose data in the literature are first described in detail and in chronological order.

In their study, Panigrahi et al. used a polymer-based e-nose system to analyze the freshness of beef stored at temperatures between 10 °C and 42 °C. They performed dimensionality reduction of the signal by applying Principal Component Analysis (PCA). The obtained data were classified by radial basis function ANN. They performed the classification processes by dividing the data they obtained into two groups as corrupted and intact, and achieved high classification accuracies [17]. Li et al. combined data from two different e-nose devices for the detection and classification of damaged apples. They used PCA for feature extraction and a probabilistic neural network for classification. They obtained results with a low error rate with the methods they suggested [18]. Cevoli et al. collected data with an e-nose containing six sensor arrays to classify cheeses according to production technique and ripening time. They tried different preprocessing methods on the data they obtained. In their study, they performed four different feature extraction algorithms and feature reduction operations with PCA, and both sets of reduced data were classified with ANN [19]. Papadopoulou et al. investigated the deterioration process of sirloins stored at different storage temperatures. In their study, the classification processes were carried out by using electronic nose data and the data obtained from microbiological analyzes. Three classes were determined as fresh, semi-fresh, and spoiled. They used the SVM method for classification and obtained high classification accuracy [20]. Dang et al. have proposed a classification system that they call an enhanced support machine community. In the study based on e-nose data, kernel PCA was used for feature extraction and a new fusion approach was proposed. They obtained high classification accuracies with their proposed methods [21]. Wijaya et al. have developed an e-nose to detect and monitor beef quality. The KNN method was used to identify four beef classes. The experimental results of the study revealed that the proposed system can distinguish beef quality with high classification accuracy [22]. Stating that wines can be distinguished according to the fermentation process, type, and year as well as the production area, Liu et al. developed an e-nose to detect different wine odors. In the experimental results of the study, where Extreme Gradient Boosting, Random Forest, SVM, and Back Propagation Neural Networks (BPNN) were used to classify the obtained data, high classification accuracies were obtained with the BPNN method [23]. Using e-nose, Ghasemi-Varnamkhasti et al. have classified different types of cheese. Various methods, such as ANN, PCA, Linear Discriminant Analysis (LDA), and SVM were used within the scope of the study. In the classification of the cheese types’ storage time, the highest accuracy was obtained with the LDA method [24]. Sarno et al. created a dataset for the detection of pork mixed with beef. By mixing seven different ratios of beef and pork, they collected data for each sample for 120 s with an e-nose. A high classification accuracy was obtained in the experimental results of the study, in which the data size was reduced by PCA and the classification was performed with SVM [25]. Wakhid et al. used different-sized chambers to determine the purity of meats and collected the data with an e-nose by placing the mixed meat in these chambers. The highest classification accuracy was obtained from the data collected in the 50 mL chamber [26]. Hibatulah et al. proposed a method that can predict the microbial population in meat. The Support Vector Machine Regression method was used in training and testing the data obtained from an e-nose. As a result of the experiments, they obtained high R^2^ and RMSE values [27].

Considering the studies in the literature, it is vital to monitor beef quality rapidly, effectively and with high accuracy. Based on these problems, this study was carried out in order to classify data collected from different cuts of meat with a higher accuracy than other studies in the literature. In addition, it is aimed to classify beef quality with high accuracy, regardless of cuts. Considering the studies in the literature and based on these aims, the procedures carried out within the scope of this study are as follows:

- E-nose data obtained from 12 different beef cuts was used.
- The data in the 12 tables created for each beef cut in the dataset were classified separately.
- The data in each of the 12 tables were reclassified with the active features determined by the ANOVA method.
- In order to eliminate the beef cut shape factor, all tables were combined into a single table.
- The 26,640 rows of data obtained as a result of combining all tables were classified by using the same methods.
- Classifications with 26,640 rows of data and features selected by ANOVA were performed with the same methods again.
- The obtained results were analyzed with confusion matrices and performance metrics.

As a result of these processes, the contributions of the study to the literature can be summarized as follows:

- The 12-table data generated for each beef cut was classified separately. In this way, the classification accuracy for each cut could be analyzed.
- Effective features were determined with ANOVA and more accurate and faster results were obtained with fewer data in each table.
- By combining all the tables in the dataset, the beef cut factor was disabled and classifications were carried out.
- With the effective features determined by ANOVA, high accuracy and high-speed prediction accuracy were achieved on all data in the dataset.

The remainder of this paper is organized as follows. Section 2 describes the material and methods, Section 3 presents the experimental results, and Section 4 provides a discussion and conclusions.

## 2. Material and Methods

**Figure 1:**
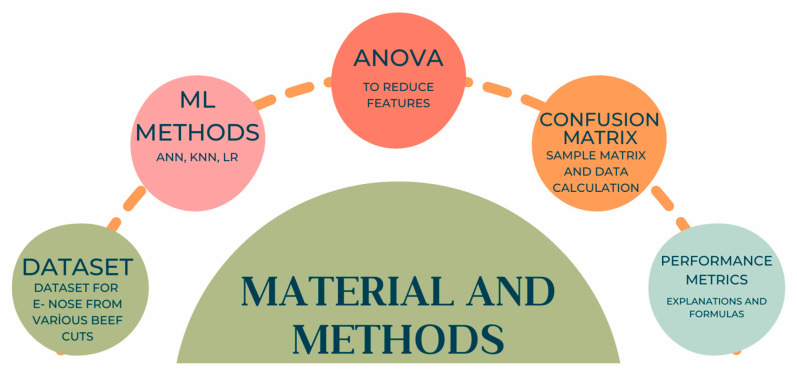
Mind map of material and methods.

In this section, the dataset used in the study, the machine learning methods (ANN, KNN, and LR), Analysis of Variance (ANOVA), which is used to reduce the number of features, increase the classification accuracy and reduce the calculation time, the confusion matrix used to observe the classification data of the classification models, and the methods utilized to measure the models’ performance are explained. A mind map showing these processes is given in Figure 1.

### 2.1. Dataset: Electronic Nose from Various Beef Cuts (ENVBC)

Wijaya et al. developed a low-cost and fast-operating meat evaluation system [28]. With the electronic nose they developed, they evaluated the variation in quality with time of different beef cuts and used 12 different beef cuts to construct the dataset. These cut shapes were round (shank), top sirloin, tenderloin, flap meat (flank), striploin (shortloin), brisket, clod/chuck, skirt meat (plate), inside/outside, rib eye, shin, and fat. The deterioration process of the beef was recorded with 11 sensors for 2220 min for each cut shape. Arduino was used for data gathering. The obtained data was constructed as a dataset consisting of 12 tables. The tables consisted of 2220 rows and 11 sensor features for each cut. For the evaluation of meat quality, 4 classes were defined: (1) excellent, (2) good, (3) acceptable, and (4) spoiled. Total viable count (TVC) values were used while determining these classes. This value indicates the number of microbial populations [29]. Table 1 shows the gas sensors used in the creation of the dataset and information on which gases these sensors can detect.

**Figure 2:**
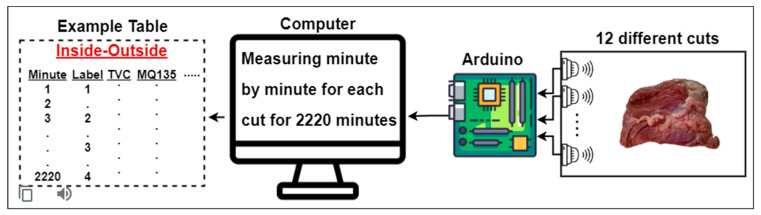
The experimental setup used to obtain the dataset [28].

**Figure 3:**
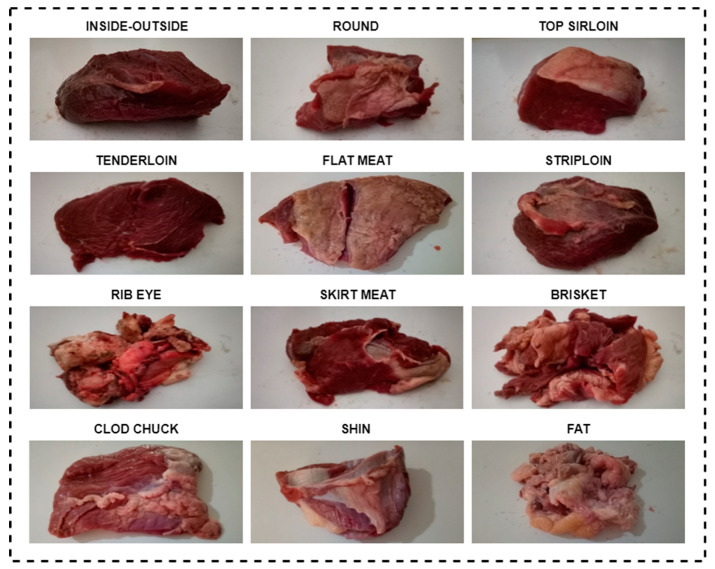
Beef cuts used in creating the dataset [28].

Figure 2 illustrates the process of obtaining data from 12 different beef cuts by sensors. Beef placed in a box was measured once per minute with 11 different sensors for 2220 min. The received data was turned into tables and a dataset consisting of 12 tables was created. The visuals of 12 different beef cuts used in the creation of the dataset are given in Figure 3.

### 2.2. Machine Learning (ML)

ML uses a combination of algorithms that parse data and then applies what it learns to make an informed decision [30]. Because machine learning uses data to feed an algorithm that can understand the relationship between input and output, it requires little human intervention after deployment [31]. Machine learning models can provide their own predictions based on the received amount of data input [32]. Moreover, they can also increase their predictive capacity as they learn more about the information they are processing. In this study, three different ML methods were used for data classification. Information about these models is given in the following sections.

#### 2.2.1. Artificial Neural Network (ANN)

An Artificial Neural Network is a network of interconnected artificial neurons, where each neuron represents an information processing unit. These interconnected nodes transmit information to each other, mimicking the human brain. The nodes interact with each other and share information. Each node receives input and performs some operations on it before transmitting [33]. These operations are performed by a nonlinear mathematical function called the activation function. The activation function transforms the input into an output that will later be used as input for other nodes. There are certain weights between the nodes and these weights are updated with each learning round of the model [34]. If the performance (accuracy) ratio is high, the weights are not updated. These weights are updated with feedback each round to improve accuracy. According to the accuracy of the result from the network, these weights are renewed through certain functions. The leftmost layer is the input layer, the last one is the output layer, and all layers in between are referred to as hidden layers. Increasing the hidden layers and the nodes within them usually increases the accuracy; however, this requires a large amount of computational power, and in some cases, increasing the number of hidden layers reduces the accuracy, contrary to expectations [35].

#### 2.2.2. K-Nearest Neighbor (KNN)

In the simplest sense, KNN is based on estimating the class of the vector formed by the independent variables of the value to be estimated. This is based on the class information of the densest nearest neighbors. The KNN (K-Nearest Neighbor) algorithm makes predictions of two fundamental values. The distance value is the distance of the point to be estimated from other points based on a Euclidean distance calculation function. The K value (neighborhood number) is determined by how many nearest neighbors the calculation will be made over, and the K value directly affects the result. If K = 1, the probability of the model being overfit is very high. If it is much greater, the model produces very general results. Therefore, estimating the optimum K value is a critical step [36].

#### 2.2.3. Logistic Regression (LR)

LR is a statistical machine learning method. It differs from linear regression by the type of line used to separate two classes. In linear regression, classification is performed by drawing a straight line, but the sigmoid function is used for classification in logistic regression. The sigmoid function compresses data between 0 and 1. Classification can be performed thanks to this function. The implementation and interpretation of the logistic regression algorithm are easy due to these features. The LR algorithm performs well if the dataset is linearly separable, i.e., it does not contain very complex data. This algorithm is less likely to have problems with overfitting. However, it can be overfit in complex and large datasets. If the number of data (number of rows) in the dataset is less than the number of features (columns), the probability of overfitting is quite high, so LR should not be used in this case [37].

### 2.3. (Analysis of Variance) ANOVA

ANOVA is a method utilized to test whether there is a statistically significant difference between the means of independent variables [38] and it is a parametric test [39]. The use of ANOVA requires certain conditions such as normal distribution of the data and equal group variances. It should not go unnoticed that the test shows only statistical differences. The difference between the variables can be detected with ANOVA; however, it cannot be determined between which variables the difference is [40]. In this study, it was determined which variables could be more effective in classification according to the data obtained from the sensors with ANOVA and TVC data. Here, ANOVA determines which features are statistically different from other features.

### 2.4. Confusion Matrix

**Figure 4:**
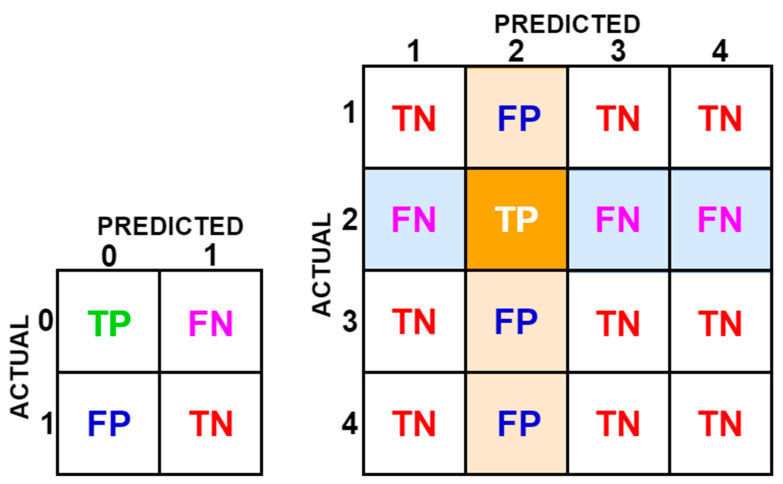
Two-class and four-class confusion matrices.

Thanks to this table, the current situation in the data set and the number of correct and incorrect predictions of the classification model can be observed. The confusion matrix contains four values for each class, regardless of its size: True Positive (TP), False Positive (FP), True Negative (TN), and False Negative (FN) [41]. True positives are TP+FN, true negatives are TN+FP. Using these data, the performance of classification models can be calculated. By closely examining the confusion matrix data in precision classification problems, the hyperparameters of the models can be adjusted, thus enabling more precise classification. The size of the confusion matrices is determined by the number of classes in the classification problem [42]. The number of classes in this study is four. Figure 4 shows TP, FP, TN, and FN values in a two-class confusion matrix and obtaining these values in a four-class confusion matrix. Obtaining values in a four-class confusion matrix is for class 2.

### 2.5. Performance Evaluation

In classification studies carried out in areas that are directly related to human health, such as health, food, and environment, the performance of the models needs to be assessed according to more than one criterion. It is extremely important to be able to build robust models, as very small increases or decreases in performance can directly affect people [43]. The performance of classification models is assessed according to various metrics. However, there are values to be necessarily checked. Accuracy (AC), F1 Score (F1), Precision (PR), Recall (RE), and Specificity (SP) metrics were used in this study. Accuracy is the percentage of samples correctly classified. F1 Score is a measure of a test’s accuracy (the harmonic mean of PR and RE), and in general, it is a measure of the precision and robustness of a model [44]. In some studies, it is possible to evaluate the results by directly giving this metric. Precision is a metric that shows how many of the positively predicted values are actually positive. Recall indicates how many of the samples that should have been predicted as positive were predicted as positive. Specificity (False Positive Rate) is a measure of how many negative data are falsely classified as positive. These metrics are calculated using the confusion matrix data [45]. The formula for each metric is shown in Table 2.

## 3. Experimental Results

**Figure 5:**
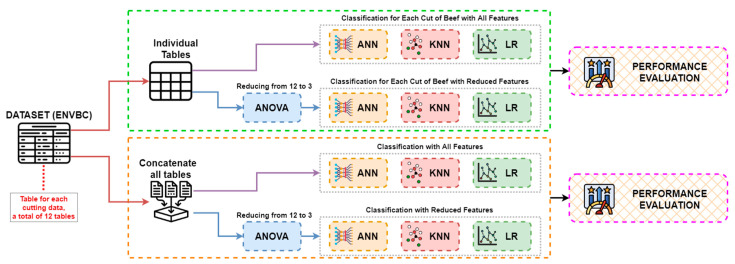
Flow chart of experimental studies.

In this section, the experimental results of the study are described. A computer with Intel^®®^ Core i7™ 12700K 3.61 GHz, NVIDIA GeForce RTX 3080Ti, and 64 GB RAM was used in performing the experiments. Coding processes were carried out with the Python programming language. The ENVBC dataset used in the study consists of a total of 12 types of tables, and each of these 12 tables is derived from measurements taken from different beef cuts. Each table has 2220 rows of data corresponding to 2220 min of measurements. A total of 11 sensors and the TVC data were provided as input to the machine learning methods, and the output predictions consisted of four classes. This dataset, in which the deterioration stages of beef are examined, needs to be classified with high success, and the data should be analyzed in detail as the results are extremely important for human health. For this reason, the data obtained from each cut were classified separately by ANN, KNN, and LR methods, and 12 different results were recorded. Following this step, feature selection was performed with the ANOVA method in order to achieve high classification accuracy and low computation time with fewer features. The same classification processes were carried out with the three features obtained, and the 12 results obtained at this stage were recorded. Then, the 12 tables were combined and a single table containing all the data was obtained in order to show that the quality of meat can be determined without considering the cut. After combining all the tables, the data were given as input to the same methods and classification results were obtained. At the last stage, reclassification processes were carried out with the features selected by ANOVA. The flow chart in Figure 5 demonstrates these processes.

**Figure 6:**
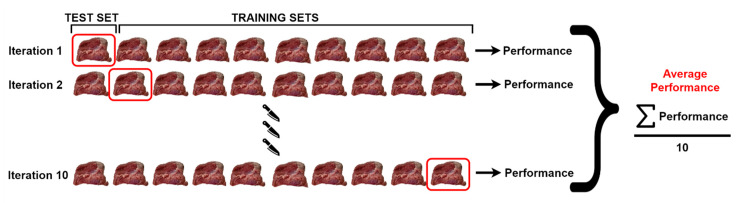
Cross-validation for this study.

The cross-validation method was used to perform detailed performance analyzes of the classification models. This method enables classification models to be compared objectively. In this method, the data set is divided into ten parts consisting of nine training parts and one test part in each cycle. The training and testing processes are carried out in as many cycles as the number of parts. The process is performed in 10 steps so that all parts are used in the test. The average classification accuracy of the model is calculated by taking the arithmetic average of the obtained results. The cross-validation structure used in the study is shown in Figure 6.

In the first stage of the classifications, 2220 rows of data in the tables created for each beef cut were given as input to the classification models, and 12 results were obtained as a result of the classifications. Table 3 gives the classification results obtained according to the beef cuts.

According to Table 3, the highest classification accuracy was obtained from the ANN model with 99.7% in the Skirt Meat and Clod Chuck beef cuts. The highest classification accuracy for each beef cut was obtained from different models. However, classification accuracy is expected to be at the highest level for each beef cut. When Table 3 is examined, it can be seen that the longest training times were achieved by the ANN model and the KNN model achieved the shortest. The longest test time was achieved by the KNN model in each use of beef cut data, and the shortest test time was achieved by LR. These times are expected to be shorter because the quality assessment of beef must be quickly carried out using the models in different environments.

In the second stage of the classifications, the selection of the effective features in each table was performed with the ANOVA method. This method determined TVC, MQ137, and MQ5 as the most effective features. Classification of data for each beef cut was carried out by using these features, and the classification results obtained are presented in Table 4.

Looking at the data in Table 3 and Table 4, it can be observed that the accuracy in classifications performed with active features has increased, and the other classification metrics have increased in line with accuracy. This proves that classification models perform better with learning. In addition, it is generally expected that the training and test times decrease in classifications carried out with a small number of features, and the obtained results show that the train and test times decreased significantly.

To evaluate the quality without considering the beef cut, all tables in the dataset were combined and a table with 26,640 rows of data was obtained. In this way, the impact of the cut shape was eliminated when evaluating the meat quality. This situation is important in terms of the practicality of the models’ use in real-life.

**Figure 7:**
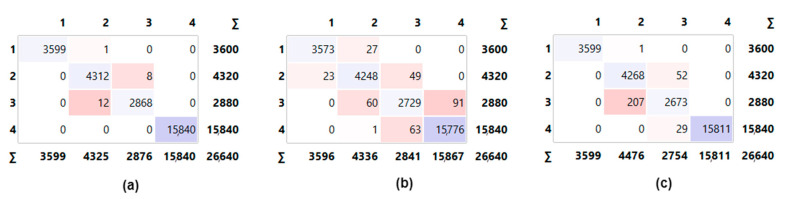
Confusion matrix of all models with concatenated data: (**a**) ANN, (**b**) KNN, and (**c**) LR.

In the third stage, classifications were carried out with all data. The confusion matrices obtained as a result of these classifications are shown in Figure 7.

Examining the confusion matrices in Figure 7, it can be seen that the correct classifications were performed with the ANN model. When evaluated according to classes, it is seen that the most correctly classified class is class 1. The performance metrics calculated by using the confusion matrix data for this classification are presented in Table 5.

In the classification carried out using all the data, the highest classification accuracy was obtained from the ANN model. In parallel with the classification accuracy metric, the highest value in other metrics was also achieved by the ANN model. Although the lowest classification accuracy was observed for the KNN model, the classification accuracy of the LR model is also very close to that of the KNN model. According to Table 5, the shortest training time was observed for the KNN model, whereas this model had the longest test time.

**Figure 8:**
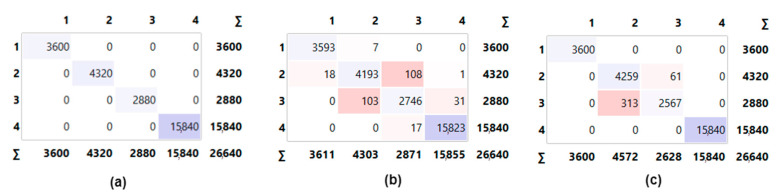
Confusion matrix of all models with concatenated data and reduced features: (**a**) ANN, (**b**) KNN, and (**c**) LR.

At the last stage of the classifications, all the data were analyzed with the active features selected by the ANOVA method. The aim here is to reduce the training and test times as well as allow the models to classify quickly. In the literature, classification accuracy does not always increase in the case of feature reduction. However, in this study, both the training and test times decreased, and the classification accuracy increased in the classifications performed with reduced features. This is an ideal situation for classification models. Figure 8 presents the confusion matrices obtained as a result of classifications by using all data and reduced features.

According to Figure 8a, all the data were classified correctly in the confusion matrix of the ANN model. The performance metrics obtained as a result of the classifications performed by using all data and reduced features are provided in Table 6.

Examining Table 6, it is seen that the ANN model achieved 100% accuracy by correctly classifying all the data. The use of active features reduced the training and test times for all models. Although feature reduction slightly affected the classification performance of the KNN model, it affected the LR model more negatively. The ANN model has come to the fore as the most preferred model among all models.

**Figure 9:**
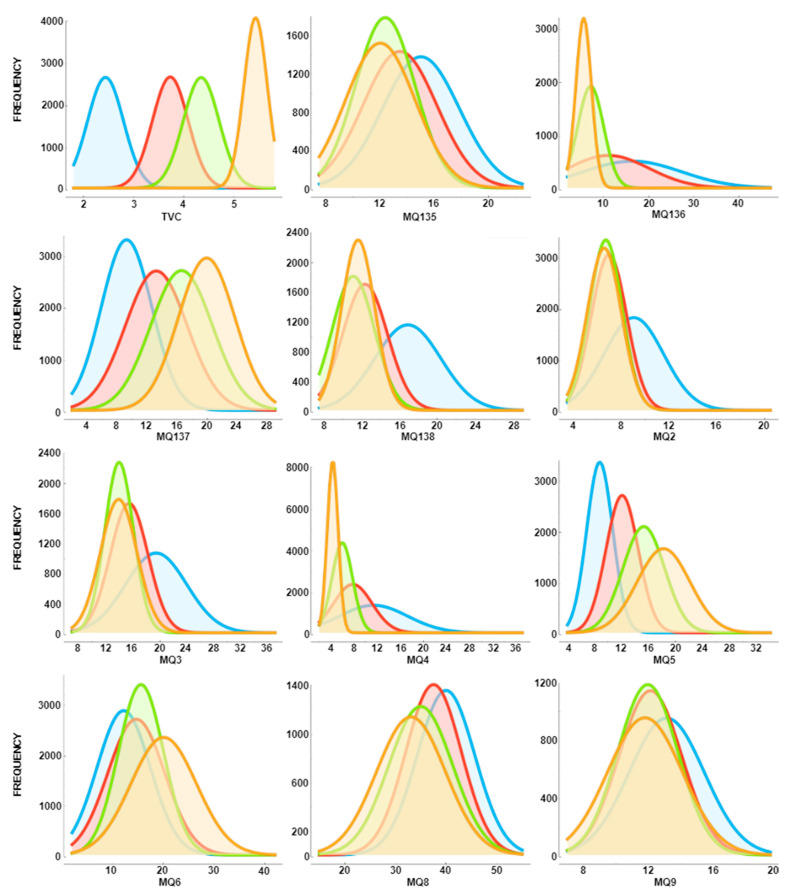
Frequencies of data by classes: 1, excellent (blue curve); 2, good (red curve); 3, acceptable (green curve); and 4, spoiled (yellow curve).

It is not surprising that the ANOVA method determines TVC, MQ137, and MQ5 features as effective. The data with these features differ according to the classes. In Figure 9, the frequencies of the feature values according to the classes in the dataset are shown.

In Figure 9, the frequencies for the TVC, MQ137, and MQ5 feature values differ according to classes. In addition, the feature values are also in different value ranges according to classes. In other features, the feature values are gathered in approximately the same area. This is a situation that complicates prediction in classification models. It also extends the training and test times of the model. The TVC, MQ137 and MQ5 features are noticeably more efficient than the other features. The effectiveness of these features was also enhanced by the ANOVA method in the study.

## 4. Discussion and Conclusions

In this study, data recorded by measuring the quality of 12 different beef cuts by sensors were used. In the dataset, there are 12 tables in total. Each table contains the data collected for each beef cut. These data were used in four different classification stages. In the first stage, each table data was given as input to the ANN, KNN, and LR models. In the second stage, among the 12 features, the most effective features were determined by the ANOVA method, which were the TVC, MQ137, and MQ5 features. Classifications were then again carried out with the same methods. 

In the third stage, all the data were combined so that the classification models were not affected by the beef cuts. The classification processes were carried out by the same models with a total of 26,640 rows of data and 12 features. In the fourth stage, the same models were used in classification processes performed with the TVC, MQ137, and MQ5 active features. As a result of these analyzes, it was observed that feature reduction processes increased the classification accuracy of the models and other performance metrics. In the classification carried out with the effective features and combined data, the ANN model achieved a classification accuracy of 100%. In addition, feature reduction was found to significantly reduce the training and test times. In the literature, there are other studies conducted with the dataset used in this study. Table 7 provides comparisons with the studies of other authors who have analyzed the dataset [28].

When Table 7 is examined, it is seen that the highest classification accuracy was achieved in this study. The classification accuracy of other studies is also reasonably high. However, since meat is an important food source for living things and can spoil very quickly, it is extremely important to determine its quality in a quick and accurate way.

The methods proposed in this study can be used to determine beef quality. The 100% classification accuracy achieved through training with 26,640 data, is a sufficient level for the classification of new data. The data can be augmented to make the models more robust. With data augmentation, the number of scenarios will increase and the models will become more informed. In addition, higher-quality sensors can be used to obtain more accurate data so that healthier decisions can be made. The proposed models are of suitable size and sufficiently fast to work in mobile applications and various embedded systems. They are also suitable for the server–client mode of operation. The data sent from the network can be quickly classified by the model on the server and sent back to the client. The obtained training and test times demonstrate that this structure is convenient.

## Figures and Tables

**Table 1:** The sensors used in the creation of the dataset and the gases they detect.

Gas Sensor	What Gases Does it Detect?
MQ2	Alcohol, LPG, smoke, propane, methane, butane, hydrogen
MQ3	Alcohol, carbon monoxide, methane, LPG, hexane
MQ4	Methane
MQ5	Alcohol, carbon monoxide, hydrogen, LPG, methane
MQ6	LPG, Propane, Iso-butane
MQ8	Hydrogen
MQ9	Methane, propane and carbon monoxide
MQ135	Nox, Alcohol, carbon dioxide, smoke, ammonia, benzene
MQ136	Hydrogen sulfide
MQ137	Ammonia
MQ138	Alcohols, aldehydes, ketones

**Table 2:** Performance metrics formulas.

Metrics	Formula
Accuracy (AC)	$\frac{TP+TN}{TP+TN+FP+FN}x100$
F1 Score (F1)	$2x\frac{PR\;x\;RE}{PR+RE}$
Precision (PR)	$\frac{TP}{TP+FP}$
Recall (RE)	$\frac{TP}{TP+FN}$
Specificity (SP)	$\frac{FP}{FP+TN}$

**Table 3:** Classification results for each cut of beef with all features.

		AC (%)	F1	PR	RE	SP	Train Time (s)	Test Time (s)
Inside-Outside	ANN	98.9	0.989	0.989	0.989	0.997	19.616	0.038
	KNN	98.5	0.985	0.985	0.985	0.995	0.104	0.124
	LR	98.7	0.987	0.988	0.987	0.996	11.948	0.012
Round	ANN	99.3	0.993	0.993	0.993	0.997	19.133	0.037
	KNN	98.8	0.988	0.988	0.988	0.994	0.106	0.11
	LR	98.9	0.989	0.989	0.989	0.995	6.114	0.012
Top Sirloin	ANN	99.3	0.993	0.993	0.993	0.998	19.668	0.039
	KNN	98.2	0.983	0.983	0.982	0.993	0.105	0.116
	LR	98.8	0.988	0.988	0.988	0.993	19.397	0.014
Tenderloin	ANN	99.4	0.994	0.994	0.994	0.998	18.746	0.036
	KNN	98.6	0.986	0.986	0.986	0.994	0.084	0.119
	LR	99.5	0.996	0.996	0.995	0.998	12.456	0.018
Flap Meat	ANN	99.5	0.995	0.995	0.995	0.998	18.143	0.036
	KNN	98.2	0.982	0.982	0.982	0.989	0.089	0.108
	LR	98.6	0.986	0.986	0.986	0.991	11.547	0.013
Striploin	ANN	99.3	0.993	0.993	0.993	0.999	18.604	0.039
	KNN	99	0.99	0.99	0.99	0.992	0.096	0.12
	LR	99.3	0.993	0.993	0.993	0.996	5.253	0.014
Rib Eye	ANN	98.7	0.987	0.987	0.987	0.997	18.53	0.038
	KNN	99.4	0.994	0.994	0.994	0.999	0.092	0.102
	LR	99.2	0.992	0.992	0.992	0.999	9.098	0.015
Skirt Meat	ANN	99.7	0.997	0.997	0.997	0.999	21.204	0.071
	KNN	99.6	0.996	0.996	0.996	0.999	0.106	0.114
	LR	99.5	0.995	0.995	0.995	0.997	9.772	0.016
Brisket	ANN	99	0.99	0.99	0.99	0.997	18.479	0.035
	KNN	98.5	0.985	0.985	0.985	0.991	0.103	0.115
	LR	98.9	0.989	0.989	0.989	0.996	11.804	0.011
Clod Chuck	ANN	99.7	0.997	0.997	0.997	0.999	20.717	0.041
	KNN	98.9	0.989	0.989	0.989	0.992	0.099	0.116
	LR	99.5	0.995	0.995	0.995	0.997	14.857	0.017
Shin	ANN	99.3	0.993	0.993	0.993	0.998	19.588	0.035
	KNN	98.7	0.987	0.987	0.987	0.994	0.114	0.126
	LR	99.2	0.992	0.992	0.992	0.999	11.946	0.016
Fat	ANN	99.5	0.995	0.995	0.995	0.997	19.733	0.037
	KNN	98.6	0.986	0.986	0.986	0.992	0.098	0.135
	LR	99.1	0.991	0.991	0.991	0.994	22.757	0.013

**Table 4:** Classification results for each cut of beef with reduced features.

		AC (%)	F1	PR	RE	SP	Train Time (s)	Test Time (s)
Inside-Outside	ANN	99.2	0.992	0.992	0.992	0.999	18.745	0.02
	KNN	99.5	0.995	0.995	0.995	0.999	0.058	0.083
	LR	96.6	0.965	0.966	0.966	0.96	6.83	0.01
Round	ANN	98.8	0.988	0.988	0.988	0.997	18.154	0.016
	KNN	99.2	0.992	0.992	0.992	0.998	0.067	0.082
	LR	99	0.99	0.99	0.99	0.997	4.818	0.007
Top Sirloin	ANN	98.7	0.987	0.987	0.987	0.998	23.533	0.018
	KNN	99.1	0.991	0.991	0.991	0.998	0.084	0.091
	LR	96	0.959	0.959	0.96	0.966	9.906	0.008
Tenderloin	ANN	99.6	0.996	0.996	0.996	0.999	17.073	0.022
	KNN	99.7	0.997	0.997	0.997	0.999	0.07	0.081
	LR	99.1	0.991	0.991	0.991	0.993	5.886	0.007
Flap Meat	ANN	99.6	0.996	0.996	0.996	0.999	17.085	0.018
	KNN	99.9	0.999	0.999	0.999	0.999	0.062	0.076
	LR	96.8	0.966	0.969	0.968	0.961	5.464	0.005
Striploin	ANN	99.9	0.999	0.999	0.999	1	18.249	0.021
	KNN	99.9	0.999	0.999	0.999	1	0.064	0.088
	LR	99.7	0.997	0.997	0.997	0.998	5.683	0.008
Rib Eye	ANN	99.5	0.995	0.995	0.995	0.999	17.062	0.02
	KNN	99.6	0.996	0.996	0.996	0.999	0.063	0.078
	LR	98.4	0.984	0.984	0.984	0.989	5.216	0.006
Skirt Meat	ANN	99.8	0.998	0.998	0.998	1	19.406	0.019
	KNN	99.5	0.995	0.996	0.995	0.999	0.073	0.091
	LR	99.4	0.994	0.994	0.994	0.998	9.594	0.01
Brisket	ANN	99.2	0.992	0.992	0.992	0.999	18.411	0.02
	KNN	98.7	0.987	0.987	0.987	0.993	0.081	0.09
	LR	97.6	0.976	0.976	0.976	0.986	7.232	0.009
Clod Chuck	ANN	99.9	0.999	0.999	0.999	0.999	19.008	0.021
	KNN	99.7	0.997	0.997	0.997	0.997	0.076	0.091
	LR	99	0.989	0.99	0.99	0.986	6.641	0.01
Shin	ANN	99.6	0.996	0.996	0.996	0.999	18.005	0.02
	KNN	99.9	0.999	0.999	0.999	1	0.081	0.088
	LR	97.7	0.977	0.977	0.977	0.977	4.342	0.006
Fat	ANN	99.3	0.993	0.993	0.993	0.999	18.875	0.019
	KNN	99.5	0.995	0.995	0.995	0.999	0.065	0.089
	LR	98.6	0.986	0.986	0.986	0.991	7.323	0.008

**Table 5:** Classification results with all features and concatenated data.

	AC (%)	F1	PR	RE	SP	Train Time (s)	Test Time (s)
ANN	99.9	0.999	0.999	0.999	1	205.253	0.154
KNN	98.8	0.988	0.988	0.988	0.994	0.493	1.368
LR	98.9	0.989	0.989	0.989	0.998	406.085	0.024

**Table 6:** Classification results with reduced features and concatenated data.

	AC (%)	F1	PR	RE	SP	Train Time (s)	Test Time (s)
ANN	100	1	1	1	1	137.617	0.129
KNN	98.9	0.989	0.989	0.989	0.997	0.205	0.0866
LR	98.6	0.986	0.986	0.986	0.997	45.535	0.011

**Table 7:** Comparisons with other studies using the same dataset.

Study	Methods	Max. Performance
Kaya et al. [46]	KNN, Linear Discriminant, Decision Tree	Accuracy: 98%
Wijaya et al. [47]	Adaboost, Random Forest, Support Vector Machine (SVM) and Decision Tree	Accuracy: 99.9%
Wijaya et al. [1]	Theoretic Ensemble Feature Selection and KNN and SVM	F1 Score: 99%
Enériz et al. [48]	ANN on FPGA	Accuracy: 93.73%
Pulluri et al. [49]	KNN, Extreme Learning Machine (ELM), SVM, ANN, Deep Neural Network (DNN)	Accuracy: 98%
Hibatulah et al. [27]	Support Vector Regression	R^2^: 0.977 and RMSE: 0.026
This Study	ANN, KNN, SVM	Accuracy: 100% Precision: 100% Recall: 100% F1 Score: 100%

## Data Availability

The dataset used in the study can be accessed from the link https://doi.org/10.7910/DVN/XNFVTS (accessed on 15 December 2022).
